# Revue systématique sur la contribution des femmes distributeurs communautaires dans la lutte contre l'onchocercose en Afrique sub-saharienne

**DOI:** 10.11604/pamj.2014.19.318.3102

**Published:** 2014-11-26

**Authors:** Marius Zambou Vouking, Carine Nouboudem Tadenfok, Christine Danielle Evina, Moustapha Nsangou, Cécile-Renée Bonono, David Yondo

**Affiliations:** 1 Centre pour le Développement des Bonnes Pratiques en Santé, Hôpital Central de Yaoundé, Yaoundé, Cameroun; 2 Délégation Régionale de la Santé Publique du Centre, Yaoundé, Cameroun; 3 Université Catholique d'Afrique Centre, Ecole des Sciences de la Santé, Yaoundé, Cameroun; 4 Université de Yaoundé I, Faculté des Arts, Lettres et des Sciences Humaines, Yaoundé, Cameroun; 5 Université de Yaoundé II, Faculté des Sciences Economiques et de Gestion, Yaoundé, Cameroun

**Keywords:** Revue systématique, contribution, femmes, distributeurs communautaires, onchocercose, Afrique sub-saharienne, systematic review, contribution, women, community distributors, onchocerciasis, Sub-Saharan Africa

## Abstract

Le Programme Africain de lutte contre l'Onchocercose (APOC) fut lancé en 1995, comme complément au brillant programme de lutte contre l'onchocercose en Afrique de l'Ouest. En 2011, plus de 80 millions de personnes ont bénéficié de cette intervention grâce à la contribution de 268 718 Distributeurs Communautaires (DC). Ses résultats significatifs occultent le rôle des femmes DC dans cette lutte cette maladie. L'objectif de cette analyse est de déterminer la contribution des femmes DC dans le traitement de l'onchocercose sous directive communautaire en Afrique sub-saharienne, et d’évaluer leur acceptation dans ledit programme. Comme stratégie de recherche, nous avons identifié toutes les études pertinentes de Janvier 1995 à Décembre 2012. Des recherches ont été effectuées dans les bases de données suivantes: Medline, Embase (*Excerpta Medica Database*), CINAHL (*Cumulative Index to Nursing and Allied Health Literature*), LILAS (*Latin American and Caribbean Literature on Health Sciences*). Nous avons également consulté les sites d'APOC et de l'OMS AFRO. Deux auteurs ont indépendamment évalué la pertinence des articles potentiels à l'aide des critères d'inclusion et d'exclusion bien définis. Six (6) des 25 études ont rempli les critères d'inclusion. Une étude a montré que 81% des populations où il y avait des femmes DC ont reçu l'Ivermectine par rapport à 78% des villages où n'exerçaient pas de femmes DC. Une autre étude a montré qu'il y a eu une prise de conscience croissante dans les IDC en faveur de l'acceptation de la participation des femmes à la distribution communautaire de l'Ivermectine. De plus, 70% des membres de la communauté interrogés dans certaines zones endémiques, ont déclaré que les femmes étaient plus engagées, persuasives et plus patientes que les hommes dans la distribution de l'Ivermectine. Au terme de cette étude, les évaluations qui ont été effectuées donnent à penser que les femmes peuvent jouer un rôle important dans la lutte contre l'onchocercose. Toutefois, des préoccupations subsistent quant aux considérations de mise en œuvre en faveur dudit rôle. Les conclusions ont aussi des implications pour la recherche future sur l’élaboration d'un cadre d’évaluation et de comparaison des interventions impliquant les DCs hommes et femmes.

## Introduction

Chaque année, les Maladies Tropicales Négligées (MTN) affaiblissent, handicapent ou tuent des millions de personnes. Elles entraînent des souffrances physiques, une stigmatisation sociale et une réduction des capacités productives [1]. Ceux qui en sont les plus affectés sont les populations les plus démunies qui vivent dans des zones rurales isolées, des bidonvilles ou des zones de conflit [1]. L'onchocercose est l'une de ces maladies, elle est provoquée par *Onchocerca volvulus*, un ver parasite transmis par certaines espèces de Simulies qui se reproduisent dans les rivières et cours d'eau rapides [2]. L'ivermectine est un microfilaricide sûr et efficace administré par voie orale depuis 1987 pour lutter contre l'onchocercose [2]. Le Programme Africain de lutte contre l'Onchocercose (APOC) fut lancé en 1995, comme complément au programme de lutte contre l'onchocercose en Afrique de l'Ouest [3]. L'APOC a été créé pour mettre en place un système durable de distribution de l'Ivermectine sous directives communautaires [3]. Les services de santé des pays Africains n’étant pas toujours capables de faire face à la forte demande de soins d'où la dépendance croissante de l'implication des communautés et de l'utilisation des volontaires pour aider à offrir les services.

En 2011, plus de 80 millions de personnes ont bénéficié de cette intervention grâce à la contribution de 268 718 Distributeurs Communautaires (DC) [2]. Ses résultats significatifs occultent le rôle des femmes DC dans cette lutte. En effet, la participation insuffisante des femmes DC a été identifiée comme une préoccupation par les partenaires de l'APOC dès le début du programme [4, 5]. De plus, les décideurs sont confrontés au défi de déterminer si l'augmentation du nombre de DC femmes a un impact sur la couverture thérapeutique de l'onchocercose. Bien que la réalisation de l’équilibre des sexes puisse avoir des avantages intrinsèques, l'impact de la responsabilisation sur la performance et les opportunités offertes aux femmes DC, est une question importante mais non encore étudiée. Dans ce cadre, une analyse de genre dans le système de santé en rapport avec les sciences sociales offre un outil d′évaluation systématique de l′influence du sexe sur le processus de participation communautaire [6]. La nécessité de la participation de la communauté, et la poursuite des engagements des décideurs et bailleurs de fonds, fournit une justification évidente pour l′analyse de genre dans les programmes de lutte contre les MTNs. Les objectifs de cette analyse sont de déterminer la contribution des femmes DC dans le traitement de l'onchocercose sous directive communautaire en Afrique sub-saharienne, et d’évaluer leur acceptation dans ledit programme. Notre but étant de donner aux décideurs africains des informations à jour basées sur des données probantes pouvant orienter leurs interventions en vue d'accélérer l’élimination de l'onchocercose en Afrique sub-saharienne.

## Méthodes

### Stratégie de recherche

Nous avons identifié toutes les études pertinentes de Janvier 1995 à Décembre 2012. Des recherches ont été effectuées dans les bases de données suivantes: Medline, Embase (*Excerpta Medica Database*), CINAHL (*Cumulative Index to Nursing and Allied Health Literature*), LILAS (*Latin American and Caribbean Literature on Health Sciences*). Nous avons également consulté les sites d'APOC et de l'OMS AFRO.

La stratégie de recherche suivante a été utilisée dans les diverses bases de données et moteurs de recherche: («contribution» ou «rôle» ET «femmes» ET «distributeurs communautaires» ou «agents relais communautaires» ET «onchocercose» ou «*Onchocerca volvulus*» ou « Ivermectine » ET «Afrique sub-saharienne» OU «pays endémique d'Afrique»). Il n′y avait aucune restriction de langue ou de statut de publication. Notre recherche s'est limitée aux dix-huit dernières années, qui correspondent au début du Programme APOC. Deux auteurs ont indépendamment évalué la pertinence des articles potentiels à l'aide des critères d'inclusion et d'exclusion bien définis.

### Critères d'inclusion

Etaient inclus dans l’étude les essais contrôlés randomisés, et non randomisés, les études controlees avant après, les etudes d'analyse des séries temporelles interrompues sur la contribution et l'impact des femmes DC dans la lutte contre l'onchocercose en Afrique sub-saharienne.

### Analyse et collecte des données

Deux auteurs ont indépendamment extrait les données et évalué les risques de biais. Les résultats statistiques des études étaient reportés et résumés. Les désaccords ont été résolus par deux autres auteurs. Les données ont été rapportées sous forme narrative.

### Evaluation de la qualité des études incluses

La qualité des études incluses n'a pas été évaluée.

### Etat actuel des connaissances

**Figure 1:**
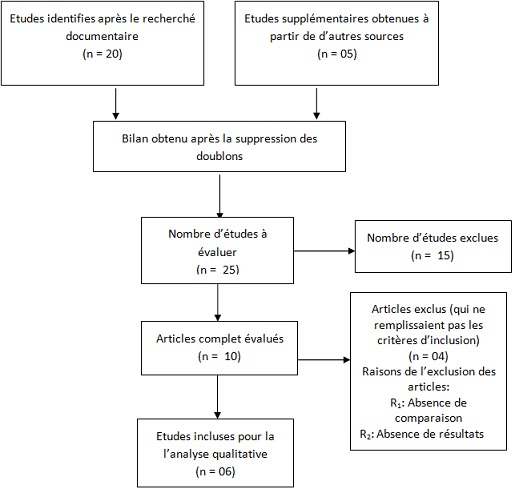
Schéma de sélection des études

Notre recherche a abouti à l'obtention de 25 études, et 06 menées en Tanzanie, au Cameroun, au Nigéria et en Ouganda ont été inclues dans la revue systématique (voir Figure 1).

### Le recrutement des femmes distributeurs communautaires

Les femmes DCs étaient soit élues par les responsables du comité de santé de l'aire [4, 7–11] soit désignés par les membres de la communauté [4, 11]. Peu d'informations sont disponibles sur leur niveau scolaire. Dans l’État d'Oyo (Nigéria), la sélection sur consensus de la communauté, des DC s'est faite sur la base de critères tels que la popularité de ces personnes, leur niveau d'instruction (école secondaire, diplôme en santé communautaire), leur honnêteté, leur éthique professionnelle, leur intérêt et leur expérience antérieure [8].

### La formation des femmes distributeurs communautaires

Les femmes DCs étaient formées annuellement à la distribution de l'Ivermectine dans le cadre du programme appelé « Interventions sous Directives Communautaires » (IDC). Elles ont également été formées à la détection des effets secondaires liées à la prise de Ivermectine et la tenue des registres [4, 7, 8, 10, 11].

### Proportion de femmes DC dans les Programmes de lutte contre l'onchocercose

Quatre études ont évalué la proportion de femmes exerçant comme DC en Tanzanie, au Nigéria, au Cameroun et en Ouganda [7, 8, 10, 11].

### Contribution des femmes distributeurs communautaires à la lutte contre l'onchocercose

L’étude menée par Brieger et collaborateurs [8] au Nigéria et au Cameroun, a montré que 81% des populations oùil y avait des femmes DC ont reçu l'Ivermectine par rapport à 78% des villages où n'exerçaient pas de femmes DC. Cette différence de trois points en faveur de l'action des femmes est un atout donc pourrait se servir les décideurs pour se rapprocher des objectifs fixés par APOC et accélérer ainsi le processus d’élimination de la maladie.

L’étude menée en Tanzanie montre qu'il a eu une prise de conscience croissante dans les IDC en faveur de la participation des femmes à la distribution communautaire de l'Ivermectine [7]. L'approche visant à intégrer la parité dans la sélection des femmes DC a augmenté la représentation des femmes ainsi que la distribution de l'Ivermectine dans les communautés concernées par les IDC [7]. En Ouganda, 70% des membres de la communauté interrogées ont déclarées que les femmes étaient plus engagées, persuasives et plus patientes que les hommes dans la distribution de l'Ivermectine. Les hommes interrogés n’étaient pas opposés à la distribution de l'Ivermectine par les femmes, au contraire, plusieurs hommes ont déclaré qu′ils seraient heureux et fiers de voir leurs épouses accepter cette tâche si celles-ci étaient désignées pour le faire [4, 11].

Dans le site de Yola (Nigéria) par contre, les femmes n'avaient pas été initialement choisies comme DCs car, dans les expériences antérieures de distribution d'Ivermectine, les DCs devaient se rendre d'une maison à l'autre, ce qui était considéré comme inapproprié pour des femmes dans cette zone musulmane [8].

Le DC est une personne sélectionnée par la communauté pour ses qualités d'intégrité, d'honnêteté, et qui est responsable de la distribution de l'Ivermectine au niveau d'une aire de santé, de faire un inventaire de cette distribution, de prendre en charge les effets secondaires simples et de référer les cas difficiles, de tenir les registres et de participer à d'autres interventions de santé [12]. Les différentes communautés ont des critères variés de sélection des DCs; généralement, la structure culturelle et politique influence la sélection et même la contribution des femmes DCs dans les programmes. D'après Amazigo [5], les leaders communautaires peuvent influencer l'inclusion des femmes comme DCs au sein des programmes de santé; de plus, la faible participation des femmes DCs dans le processus de décision et de participation communautaire est à l'origine de la faible couverture de la distribution de l'Ivermectine dans la plus part des zones endémiques d'Afrique. Lorsque les DCs n'avaient pas été choisis par la communauté dans le cadre d'un vaste processus consensuel, des problèmes d'allégeance à divers clans ou de manque de dévouement à la communauté se sont fait jour au fil du temps [3]. Les femmes DCs choisies par la communauté sont donc plus respectées, et disposées à travailler dans les conditions déterminées par la communauté et ont donc un niveau plus élevé de rétention.

Le mandat du Programme APOC consiste à mettre en place d'ici à 2015 un dispositif dirigé par les pays pour éliminer l'onchocercose en tant que problème de santé publique [2]. Les résultats des études ont montré qu'une proportion importante des personnes traitées (environ 61%) était des femmes, bien que seulement 24% des DCs étaient des femmes. Ce déséquilibre contacté dans le ratio malades/DCs montre l'ampleur du travail qu'il reste à faire dans la lutte contre cette maladie. Les raisons de cette disparité ont trait en grande partie aux barrières culturelles et religieuses. En effet, il reste des régions de la Tanzanie où les DCs sont encore à prédominance masculine, où les responsables communautaires ont décidé de cela eux-mêmes [7] ou bien où il est culturellement mal vu et dangereux pour les femmes d'assumer un rôle qui les amène à se rendre sans escorte, dans les maisons d'autres personnes comme dans le nord du Nigéria [8].

Cependant, bien que la réalisation de l’équilibre des sexes puisse avoir des avantages, cette stratégie va à l'encontre des IDCs et, bien que le programme APOC puisse encourager les communautés en mettant en exergue l'importance des questions de genre, il revient en fin de compte aux communautés de désigner ceux qu'ils jugent les plus aptes pour jouer ces rôles.

Lors de la Conférence mondiale sur les femmes de Beijing de 1995, les Gouvernements se sont engagés à intégrer la problématique hommes-femmes dans l'ensemble des processus d’élaboration des politiques et de planification du développement ainsi que des processus de mise en œuvre et d’évaluation de ces politiques et programmes [13]. L’étude menée par Brieger et collaborateurs au Nigéria et au Cameroun [8], a montré que 81% des populations oùil y avait des femmes DCs ont reçu l'Ivermectine par rapport à 78% des villages où n'exerçaient pas de femmes DCs. Cette différence de trois points en faveur de l'action des femmes est un atout donc pourrait se servir les décideurs pour se rapprocher des objectifs fixés par APOC et accélérer ainsi le processus d’élimination de la maladie.

Des dispositions ont d'ailleurs été prises pour apporter une aide financière aux femmes DCs depuis 2009 [1]. De plus, l'une des recommandations d'APOC aux responsables de Projets est de solliciter l′aide des chefs religieux dans les zones musulmanes pour résoudre le problème de genre parmi les DCs [1]. Le rapport 2010 les IDC de fait état de ce que certains membres des communautés ont jugé préférable de faire exécuter certaines interventions comme la distribution des antipaludéens par des femmes alors que d'autres étaient plus souvent confiées à des hommes [1].

La revue systématique de Douthwaite et collaborateurs a montré le rôle important qu'ont joués les femmes agents de santé communautaires dans l'utilisation des méthodes contraceptives au Pakistan [14]. Le rapport d’évaluation de 2012 sur le niveau d'atteinte des Objectifs du Millénaire pour le Développement (OMD) indique également le lien étroit entre l'atteinte de l'objectif numéro 3 sur la réalisation des objectifs 4 et 5 qui ont trait, respectivement, à la santé juvénile et maternelle car l'autonomisation sociale, économique et politique des femmes que cherche à obtenir l'objectif 3 est importante pour garantir la survie des femmes et des enfants [15].

### Limites de l’étude

Cette revue systématique comporte plusieurs limites potentielles. Tout d′abord, il est possible que certaines études publiées sur le sujet n'aient pas été retrouvées lors de la recherche documentaire. Toutefois, des efforts considérables ont été faits pour identifier des études supplémentaires en parcourant les listes de référence de la littérature identifiée. Deuxièmement, notre définition des DCs peut avoir exclu certaines catégories de personnes impliquées dans la distribution de l'Ivermectine. Enfin, le manque d′uniformité dans les plans d′étude et les rapports consultés ont rendu impossible l’évaluation comparative de leur qualité.

## Conclusion

Au terme de cette évaluation, les résultats de certaines etudes ont identifié la sous-utilisation des femmes DC comme l'une des raisons de l'efficacité limitée ou, dans certains cas, l’échec pur des interventions liées à la distribution de l'Ivermectine dans la lutte contre l'onchocercose en Afrique subsaharienne. Les évaluations qui ont été effectuées donnent à penser que les femmes peuvent jouer un rôle important dans la lutte contre l'onchocercose. Toutefois, des préoccupations subsistent quant aux considérations de mise en œuvre en faveur du dit rôle. Les conclusions ont aussi des implications claires pour la recherche future sur l’élaboration d'un cadre d’évaluation et de comparaison des interventions de santé publique impliquant les DCs hommes et femmes.
